# Robot Grasp Planning: A Learning from Demonstration-Based Approach †

**DOI:** 10.3390/s24020618

**Published:** 2024-01-18

**Authors:** Kaimeng Wang, Yongxiang Fan, Ichiro Sakuma

**Affiliations:** 1 FANUC Advanced Research Laboratory, FANUC America Corporation, Union City, CA 94587, USA; 2 Department of Precision Engineering, The University of Tokyo, Tokyo 113-8654, Japan; sakuma@bmpe.t.u-tokyo.ac.jp

**Keywords:** grasp synthesis, learning from demonstration, skill transfer, robot learning

## Abstract

Robot grasping constitutes an essential capability in fulfilling the complexities of advanced industrial operations. This field has been extensively investigated to address a range of practical applications. However, the generation of a stable grasp remains challenging, principally due to the constraints imposed by object geometries and the diverse objectives of the tasks. In this work, we propose a novel learning from demonstration-based grasp-planning framework. This framework is designed to extract crucial human grasp skills, namely the contact region and approach direction, from a single demonstration. Then, it formulates an optimization problem that integrates the extracted skills to generate a stable grasp. Distinct from conventional methods that rely on learning implicit synergies through human demonstration or on mapping the dissimilar kinematics between human hands and robot grippers, our approach focuses on learning the intuitive human intent that involves the potential contact regions and the grasping approach direction. Furthermore, our optimization formulation is capable of identifying the optimal grasp by minimizing the surface fitting error between the demonstrated contact regions on the object and the gripper finger surface and imposing a penalty for any misalignment between the demonstrated and the gripper’s approach directions. A series of experiments is conducted to verify the effectiveness of the proposed algorithm through both simulations and real-world scenarios.

## 1. Introduction

In recent years, there has been a notable escalation in the demand for industrial robots, driven by the need to enhance productivity and boost product quality. With the continuous advancements in robotics technology, the trend toward increased robot installations has been significantly pronounced in sectors such as logistics and assembly production. Among the broad spectrum of robotics research, the capability of robot grasping stands out as a fundamental skill that is crucial for the execution of complex tasks in industrial environments [1]. Despite being a subject of research for numerous decades, robot grasp planning in the context of industrial automation presents persistent challenges due to the lack of understanding of the constraints that are inherent to the downstream processes in industrial operations [2].

To address this challenge, learning from demonstration (LfD) has emerged as a promising approach that synthesizes robot grasping by observing human demonstrations. LfD offers an intuitive and straightforward method for non-experts in robotics to program a new task on a robot. Despite significant research efforts dedicated to efficiently transferring human grasping skills to robots, substantial challenges remain due to the low complexity and dexterity of the robot gripper compared to the human hand, as well as the diverse array of object shapes encountered in practical applications. Recent advancements in data-driven methods have made remarkable progress in the extraction of grasping skills by leveraging deep learning techniques. However, these methods typically require a large amount of data for training, leading to time-consuming and labor-intensive processes [3]. On the other hand, traditional analytic approaches formulate grasp planning as an optimization problem, focusing on achieving a stable grasp. The combination of human intention with optimization techniques enables robots to execute functional grasps while being mindful of constraints emanating from downstream processes. Existing methods typically represent human grasping skills through a contact model and then search for an optimal grasp that aligns with the corresponding contact model based on prior knowledge of object shapes and gripper configurations [4]. However, a notable limitation of many current approaches is their reliance on pre-defined grasping taxonomies, which aim to bridge the gap in configuration differences between human hands and robot grippers. This pre-defined grasping taxonomy hinders the ability to generalize across a broad spectrum of industrial tasks.

In this work, we propose a novel LfD-based grasp-planning approach, which incorporates human grasp skills into an optimization framework to synthesize a stable grasp. This method revolves around extracting key elements of human grasp skills, namely the contact region and the approach direction, from a single human demonstration. The key idea of our approach is to account for high-level task knowledge while addressing the difference in configuration between human hands and robot grippers. More specifically, the proposed method utilizes contact regions as a foundational element and applies optimization to reduce the surface fitting error between the robot gripper fingers and the object. To achieve a functional grasp, both the contact region and the approach direction are crucial, as often observed in human grasp skills [5]. To extract this information from human demonstrations, we propose an intuitive and straightforward method that involves passing a plane formed by the thumb and index finger through the object. The obtained grasp skills are then fed into an optimization algorithm to generate a grasp that closely approximates the human demonstration. In summary, our main contributions are:

- We propose a novel LfD-based grasp-planning framework that utilizes both the contact regions and approach direction as key skills derived from a single human demonstration. This approach effectively harmonizes human intention with the environmental constraints of both the objects and the robot gripper.
- We develop an intuitive and straightforward method for detecting the contact regions and approach direction by employing a specific hand plane formed by the thumb and index fingers using a single RGB-D camera.
- We integrate human grasp skills, encompassing both the contact regions and approach direction, into an optimization problem. This integration aims to generate a stable functional grasp that aligns with human intention while considering environmental constraints.

We note that a short conference version of this work was presented in [6]. Our initial conference paper did not delve into specific challenges such as collision avoidance and internal grasp complexities. This manuscript addresses these critical aspects, supplemented by additional analytical experiments to provide deeper insights.

## 2. Related Works

Data-driven methods: The data-driven approach in robot grasping has gained considerable traction due to its ability to learn grasp features from a large amount of data by leveraging deep learning techniques [7]. Benefiting from robustness to perception errors and the capacity to generalize to unseen objects, this approach has been successfully deployed in various applications, notably in bin-picking scenarios [8]. However, the collection of large volumes of training data is a costly and resource-intensive endeavor. Additionally, generating a feasible functional grasp in complex industrial settings often requires an understanding of environmental constraints and downstream procedures, an aspect that data-driven methods may struggle to incorporate effectively. In response to the challenges associated with data collection, Reinforcement Learning (RL) methods have emerged as a potential solution. RL methods enable robots to automatically learn grasping policies through self-supervised interaction with the environment [9]. The advent of advanced computer graphics has further facilitated this process, allowing researchers to train grasping policies in simulation environments and then transfer them to real-world applications [10]. However, setting up training simulations can be daunting for novice users. Moreover, due to the difficulty in acquiring general skills applicable across a variety of tasks, these methods often require extensive retraining for different task configurations. In contrast, the proposed method synthesizes the functional grasp by observing human demonstration once, without requiring extensive data collection and time-consuming training processes.

Analytic methods: Analytic approaches in robot grasping search for a stable grasp by formulating and solving an optimization problem, utilizing prior knowledge of object shapes and gripper configurations [11]. These methods are suitable for well-structured industrial environments since they are low data-dependent and eliminate the need for tedious supervised or unsupervised training. Some analytic strategies represent a grasp as a set of contact points, sampling gripper poses in the configuration space to seek an optimal grasp. This process often employs various metrics, such as contact surface matching [12] or gripper configuration [13]. However, these approaches do not always guarantee that the generated grasps can successfully perform specific post-grasp motions in line with the downstream processes. Alternative approaches focus on developing a taxonomy of grasp types tailored for different manipulation purposes. This type of approach can initially assign a feasible functional grasp and enhance sample efficiency by reducing the complexity of the search space [14]. An in-depth analysis of grasp taxonomy for continuum robots was presented in [15]. Based on this grasp taxonomy, the study in [16] introduced an analytical grasp synthesis approach, allowing continuum robots to adapt grasping strategies to diverse objects and tasks. In [17], a comprehensive taxonomy of human grasp types was presented through an analysis of the data recorded from 40 healthy subjects performing 20 unique hand grasps. Extending this concept, ref. [18] proposed an approach to predict the best grasp type from a taxonomy of 33 grasp classes in multi-object scenarios using just a single RGB image. However, the taxonomy-based approach is heavily dependent on predefined shape priors of both grippers and objects, which can limit its generalization capabilities for unseen objects. In contrast, our approach directly transfers human grasp skills to the robot through observations of human demonstrations, without the need for a predefined taxonomy of grasp types, which enables a more flexible and adaptive approach to robot grasping.

Grasp synthesis by learning from demonstration: The remarkable grasping skills of humans, capable of handling a diverse range of objects in complex task contexts, have sparked significant interest in the field of robotics, particularly in the transfer of these skills to robots, as surveyed in [19]. To recognize the human grasping pose, many different methods have been discussed. Early research in the field predominantly relied on a data glove for the precise detection of human finger positions, which were then replicated by robotic grippers. A teleoperation system utilizing a data glove to control a multi-fingered robotic hand was developed in [20]. The integration of tactile sensors into this glove-based framework plays a crucial role in understanding how the hand interacts with different objects. In [21], a data glove equipped with multiple Inertial Measurement Units (IMUs) and tactile sensors was designed to capture the complex dynamics of hand–object interactions in real time. However, the complexity of data gloves, especially in integrating various sensors, presents significant challenges in terms of cost and maintenance. To simplify the system of human grasping demonstration, the study in [22] proposed the use of thermal sensors to trace contact areas. With the advent of deep learning technologies, more recent studies have begun to employ simple cameras to track 3D hand poses [23]. A learning from demonstration pipeline designed to infer the poses of both the human hand and the object and then transfer these relative poses into robot grasping instructions is discussed in [24]. The integration of human demonstrations into the initialization of grasping policies has been shown to significantly improve sample efficiency for both reinforcement learning and heuristic-based methods. This efficiency is achieved by limiting the search space to a more constrained domain. A critical element in learning from demonstration (LfD) approaches is the contact model between the human hand and the object, which was extensively explored in [25]. Some researchers measure accurate contact points between the fingertips and objects through the use of a specific data glove and then map the contact points onto the corresponding robot gripper fingers [26,27]. Although these methods provide an intuitive way to transfer human grasping poses to robots, the reliance on single-point approximations imposes rigid mapping constraints and sacrifices flexibility in accounting for the configuration differences between human hands and robot grippers. To relax the mapping constraints, recent research has shifted its focus toward contact regions, recognizing that human grasps typically involve surface matching between the hand and the object [28]. However, most of these approaches employ specialized equipment (e.g., thermal cameras) or manual labeling to identify the contact regions, often overlooking the importance of the grasping approach direction, which could further narrow the search space for optimal grasping. In contrast, our method proposes a novel approach to detect both the contact regions and approach direction using a single RGB-D camera, a common device in industrial settings. In addition, the proposed optimization method formulates the surface fitting issue, aiming to minimize the distance between the demonstrated contact regions and gripper finger surface, as well as the misalignment between the demonstrated approach direction and the robot gripper, as a least-squares problem to accelerate the computation required for grasp searching.

## 3. Proposed Approach

**Figure 1:**
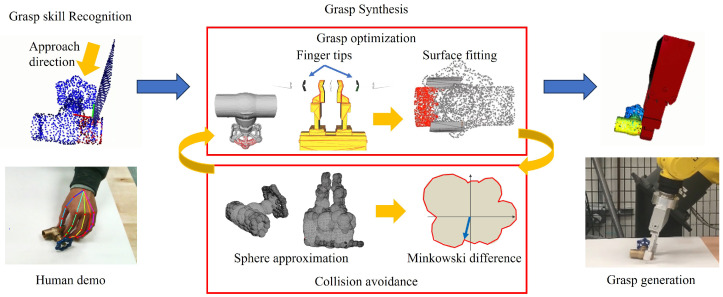
The pipeline of the proposed approach consists of two stages: grasp skill recognition and grasp synthesis. First, the contact regions and approach directions are recognized from the human demonstration. Then, grasp optimization, which iteratively fits the surface between the gripper finger surface and the contact region, is employed to synthesize the grasp corresponding to the human demonstration. The generated grasp pose is evaluated to ensure it is collision-free. If a collision is detected, the gripper is moved to a safe pose, and the grasp optimization process is repeated to generate a new pose.

The proposed method for robot grasp synthesis is structured into two stages: grasp skill recognition from human demonstration and grasp synthesis with the optimization method, as shown in Figure 1. The process starts with the identification of contact regions and approach directions from a human demonstration. This identification involves defining a hand plane formed by the thumb and one of the other four fingers [29]. The approach direction is determined by combining the directions of the thumb and index finger. The contact region on the object is then determined by intersecting this hand plane with the object’s surface and selecting proximate surface points. Following the extraction of the contact region and approach direction, the grasp optimization process, which iteratively fits the surface of the gripper fingers to the previously identified contact region and aligns the direction of the gripper approach with the human demonstration, is employed to determine the best grasping pose. The optimization is mathematically formulated as a least-squares problem, taking into account the grasp skills extracted from the human demonstration. Once a grasp pose is generated, it undergoes a critical evaluation to ensure it is collision-free. If a collision is detected, the gripper is moved to a safe pose, and the grasp optimization process is repeated to generate a new collision-free pose.

### 3.1. Grasp Skill Recognition

**Figure 2:**
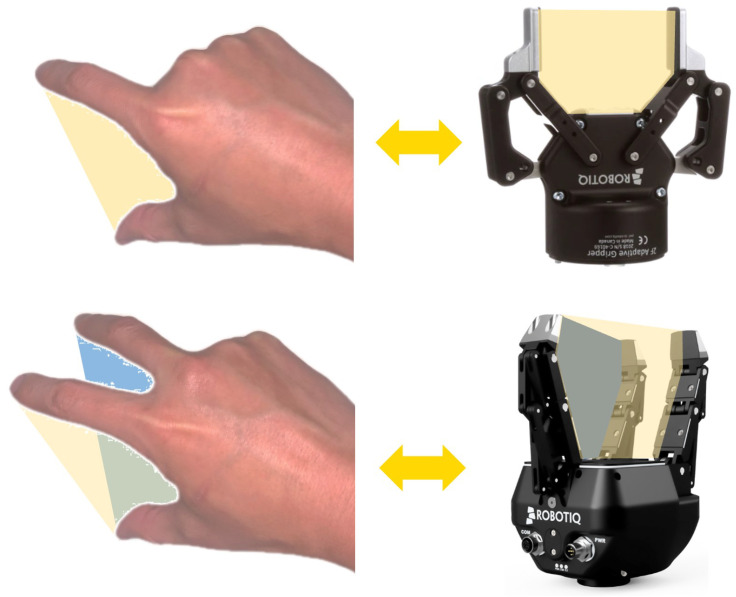
The hand-plane definitions in the two- and three-finger Robotiq gripper cases.

**Figure 3:**
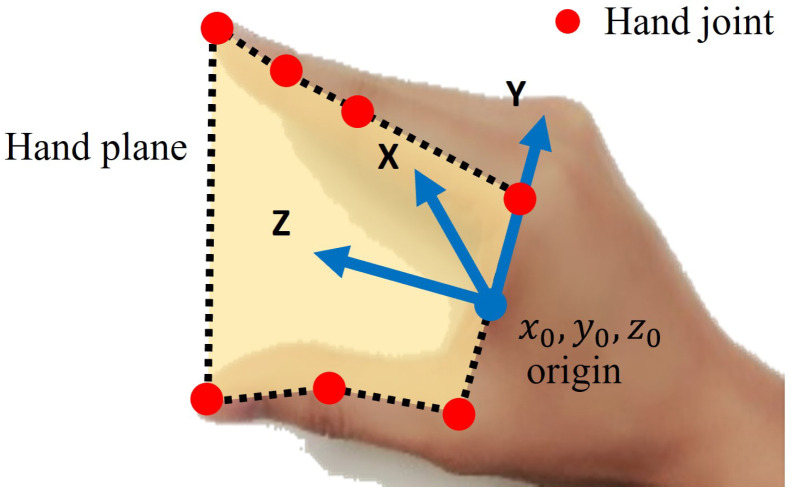
The hand plane is formed by fitting hand joints on the thumb finger and index finger.

During the human demonstration phase, the movements of the human hand are tracked by leveraging deep learning techniques [30], employing a commercial camera. The research in [31] has proven that the majority of human grasp poses are formed by the thumb and some other opposing fingers. This finding has significant implications for robot gripper design, many of which mimic this fundamental grasp characteristic by incorporating a thumb finger and a set of opposing fingers. Therefore, in this work, the hand plane is defined by the thumb and one of the other four fingers. As shown in Figure 2, one hand plane is defined by the thumb and index fingers in the case of a parallel gripper, and two hand planes for a three-finger gripper are defined: one formed by the thumb and index fingers, and another formed by the thumb and middle fingers. The coordinate system of the hand plane is calculated based on the 3D positions of specified hand joints on the thumb and index fingers, as shown in Figure 3. The midpoint between the root joints of the thumb and index fingers is defined as the origin of the hand-plane coordinate system. The normal vector of the hand plane is defined as the X-axis, whereas the Y-axis is determined by the vector connecting the thumb and index root joints. The Z-axis is then established following the right-hand rule.

The approach direction, a crucial feature for functional grasp, indicates grasp reachability. Upon determining the hand plane, the Z-axis direction is selected as the approach direction, a concept similarly explored in [5]. Note that the approach direction is uniformly calculated using the hand plane formed by the thumb and index fingers, regardless of the number of fingers on the gripper.

Our method adopts a novel approach to determining the contact region for robot grasping. Instead of striving for pinpoint accuracy in detecting the positions where human fingertips contact the object surface, the hand plane is utilized to estimate a broader contact region on the object. This strategy not only simplifies the process but also enhances the robustness of our method, particularly in optimizing the grasping pose and accommodating the configuration differences between the human hand and the robot gripper.

**Figure 4:**
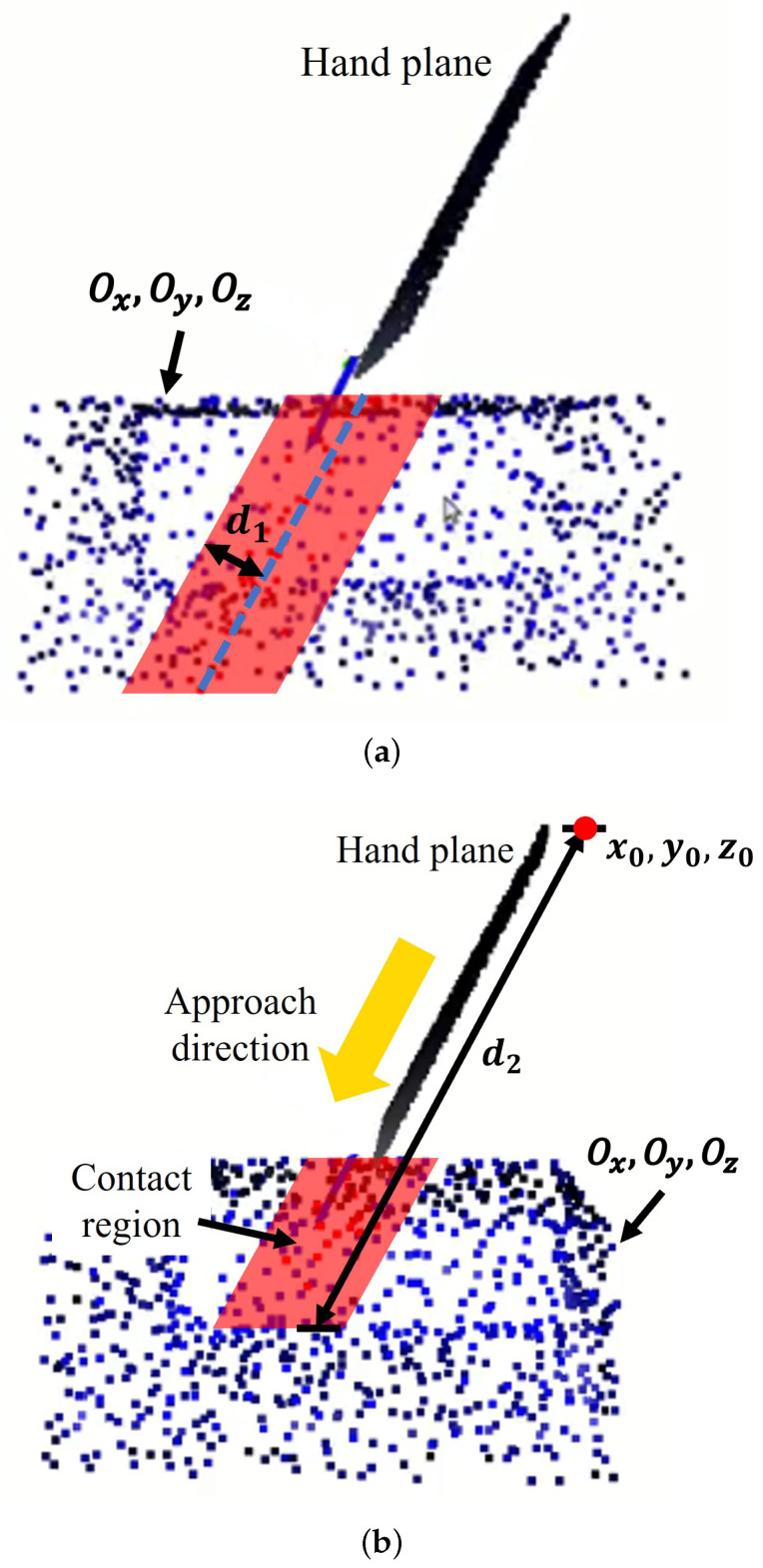
The contact region is determined by passing the hand plane through the object’s point cloud (blue). (**a**) First, select the points (red) within the distance $d_{1}$ to the hand plane. (**b**) Second, select the points (red) from the first step within the distance $d_{2}$ to the origin of the hand plane.

Figure 4 exemplifies the process of identifying the contact region on an object. To calculate this contact region, we employ a two-step procedure. The first step involves passing the hand plane through the point cloud of an object. During this phase, all points on the object surface that fall within a specified tolerance distance, denoted as $d_{1}$, from the hand plane are selected using Equation (1). These points serve as preliminary candidates for the contact region.

(1) $$\frac{|AO_{x}+BO_{y}+CO_{z}+D|}{\sqrt{A^{2}+B^{2}+C^{2}}}\leq d_{1}$$

where A, B, C, and *D* are the coefficients defining the hand plane Ax+By+Cz+D=0. Each point within the object’s point cloud is denoted by its 3D coordinates $O_{x},O_{y},$ and $O_{z}$. The parameter $d_{1}$ is the first tolerance distance, which represents the permissible distance from any point in the object’s point cloud to the hand plane. This tolerance distance is determined based on the width of the gripper finger and subsequently guides the selection of the contact area. The results of the first step are shown in Figure 4a. The selected volume contains points from the object’s point cloud that fall within the tolerance distance $d_{1}$ from the hand plane.

In the second step, we refine the selection of potential contact points on the object that were initially identified in the first step. This refinement involves selecting only those points that are within a second tolerance distance, denoted as $d_{2}$, from the origin of the hand plane using Equation (2). The purpose of this second tolerance distance is to ensure that the chosen contact regions on the object are not too far away from the demonstrated grasp pose.

(2) $$\sqrt{{\left(O_{x}-x_{0}\right)}^{2}+{\left(O_{y}-y_{0}\right)}^{2}+{\left(O_{z}-z_{0}\right)}^{2}}\leq d_{2}$$

where $x_{0},\;y_{0},\;z_{0}$ denotes the origin of the hand-plane coordinate, and the parameter $d_{2}$ is the second tolerance distance, which is determined based on the length of the gripper finger. The results of the second step are shown in Figure 4b. Only the points within the object’s point cloud, where the distance to the origin of the hand-plane frame does not exceed the specified threshold $d_{2}$, are retained.

### 3.2. Grasp Synthesis

#### 3.2.1. Grasp Optimization

The contact regions on the object that align with the human demonstration are identified and then employed in an optimization computation. It is generally observed that a larger contact surface between the gripper and the object results in greater friction, which, in turn, facilitates the formation of a more robust grasp. Leveraging this principle, an iterative surface fitting (ISF) optimization method, adapted from the Iterative Closest Point (ICP) algorithm, was proposed in [32].

Compared with the conventional ICP approach, the ISF method deforms the gripper fingertip surfaces while obeying kinematic constraints and concurrently aligns these modified surfaces with the target object’s surface. This method is more precisely formulated in Equation (3).

(3) $$\begin{array}{cc} \min_{R,t,\delta d}\; & \left(E_{p}+\lambda E_{n}\right)\\ s.t.\; & E_{p}\left(R,t,\delta d\right)=\sum_{i=1}^{m}\sum_{j=1}^{2}{\left({\left(p_{i_{j}}-q_{i_{j}}\right)}^{T}n_{i_{j}}^{q}\right)}^{2}\\ \; & E_{n}\left(R\right)=\sum_{i=1}^{m}{\left({\left(Rn_{i}^{p}\right)}^{T}n_{i}^{q}+1\right)}^{2} \end{array}$$

where the rotation *R* and translation *t* are the transformation matrix from the robot gripper to the object, and δd is the displacement between the gripper fingers. The variables $p_{i}$, $q_{i}$, $n_{i}^{p}$, and $n_{i}^{q}$ represent the points on the robot gripper surface, the corresponding point on the object surface, the normal vector at point $p_{i}$ on the gripper surface, and the normal vector at point $q_{i}$ on the object surface, respectively. The variable *m* is the total number of points, and j=1,2 represents the indexes of two fingers. According to [32], Equation (3) can be further formulated as a least-square problem $\min_{R,t,\delta d}\;{∥Ax-b∥}^{2}$.

The core objective of the ISF method is to search for an optimal transformation matrix (rotation *R* and translation *t*), as well as the displacement (δd) of the gripper fingers, by minimizing the surface fitting error. In practical terms, the most intuitive approach involves initializing R,t in Equation (3) with the pose corresponding to the human grasp. This initialization forms the baseline from which the ISF algorithm iteratively computes the errors $E_{p}$ and $E_{n}$ to refine the fit to the contact surface. However, this method may diverge from the initial human grasp pose due to the difference in fingertip shape between human hands and robot grippers. To address this, we incorporate the approach direction derived from the human demonstration into the optimization process as a constraint in Equation (4). This incorporation ensures a more accurate and feasible replication of human-like grasping by the robot.

(4) $$\begin{array}{c} \min_{R,t,\delta d}\;{∥Ax-b∥}^{2}\\ s.t.\;Rn_{z}=n_{app} \end{array}$$

where $n_{z}$ denotes the Z-axis direction of the gripper, and $n_{app}$ represents the approach direction, determined from the human demonstration. The rotation matrix *R* is designed to align $n_{z}$ with $n_{app}$.

To effectively solve this optimization problem while maintaining the integrity of the approach direction constraint, as defined by the human demonstration, Equation (4) is rewritten as follows:

(5) $$\min_{R,t,\delta d}{\;∥Ax-b∥}^{2}+\omega^{2}{∥\left(Rn_{z}\right)\times n_{app}∥}^{2}$$

where $\omega^{2}$ is a weight parameter to balance the surface matching accuracy and the alignment of the approach direction. Under the assumption that the rotation angle involved in each iterative step is small, it becomes feasible to approximate the rotation matrix *R* as follows:

(6) $$R\approx \left[\begin{array}{ccc} 1 & \delta \psi & \delta \theta \\ \delta \psi & 1 & -\delta \phi \\ -\delta \theta & \delta \phi & 1 \end{array}\right]=I+\hat{\delta r}$$

where $\hat{\delta r}\in so\left(3\right)$ is the skew-symmetric matrix form of the rotation vector $\hat{\delta r}=\left[\delta \phi ,\delta \theta ,\delta \psi \right]$, which contains the small rotational changes in three dimensions—roll (δϕ), pitch (δθ), and yaw (δψ). By substituting Equation (6), which characterizes the skew-symmetric matrix $\hat{\delta r}$, into Equation (5), the second term of Equation (5), which concerns the alignment of the approach direction, can be rewritten as follows:  

(7) $$\begin{array}{cc} \; & \omega^{2}{∥\hat{n_{app}}\cdot \hat{n_{z}}\cdot \delta r+\hat{n_{z}}\cdot n_{app}∥}^{2}\\ = & \omega^{2}{∥E\cdot \delta r-f∥}^{2}\\ \mathrm{where}\; & E=\hat{n_{app}}\cdot \hat{n_{z}},\;f=-\hat{n_{z}}\cdot n_{app} \end{array}$$

Consequently, by integrating Equation (7) into Equation (5), the optimal grasp pose can be calculated as

(8) $$\begin{array}{cc} \; & \left[\delta r,t\right]={\left(A^{T}A+\omega G^{T}G\right)}^{-1}\left(A^{T}b+\omega G^{T}F\right)\\ \mathrm{where}\; & G={\left[E^{T},0_{3\cdot 3}^{T}\right]}^{T},\;F={\left[f^{T},0_{3}^{T}\right]}^{T} \end{array}$$

Using Equation (8), the optimization problem is formulated into a standard least-squares problem that can be solved efficiently. The optimal finger relative displacement is calculated using Equation (9), given by [32].

(9) $$\begin{array}{c} \delta d^{*}=\frac{\sum_{i=1}^{m}\sum_{j=1}^{2}K_{i_{j}}H_{i_{j}}}{\sum_{i=1}^{m}\sum_{j=1}^{2}K_{i_{j}}^{2}}\\ s.t.\;\delta d^{*}\in \left[d_{min},d_{max}\right] \end{array}$$

where $K_{i_{j}}=0.5{\left(-1\right)}^{j-1}{\left(Rv\right)}^{T}n_{i_{j}}^{q}$, and $H_{i_{j}}={\left(Rp_{i_{j}}+t-q_{i_{j}}\right)}^{T}n_{i_{j}}^{q}$. $d_{min}$ and $d_{max}$ indicate the minimum and maximum distances between two fingers, and *v* is the direction vector of the finger movement.

The pseudo-code of the proposed LfD-based iterative surface fitting is summarized in Algorithm 1. The proposed method is designed to optimize for the optimal gripper transformation $R^{*},t^{*}$ with fixed δd. Concurrently, the finger optimization focuses on determining the optimal finger displacement $\delta d^{*}$ while maintaining fixed values for R,t.
**Algorithm 1** Learning from demonstration-based iterative surface fitting
1:**Input: **$p_{i},n_{i}^{p}\in gripper,q_{i},n_{i}^{q}\in object$2:**Output: **$R^{*},t^{*},\delta d^{*}$3:**Init: **$R^{*}=R_{handplane},t^{*}=t_{handplane},\delta d^{*}=0,err=\infty$4:**while $err-E(R^{*},t^{*},\delta d^{*})>▵err$ do**5:     $R^{*},t^{*}\leftarrow min_{R,t}E\left(R,t,\delta d^{*}\right)$ using Equation (8)6:     $\delta d^{*}\leftarrow min_{\delta d^{*}}E\left(R^{*},t^{*},\delta d\right)$ using Equation (9)7:     $err\leftarrow E(R^{*},t^{*},\delta d^{*})$ using Equation (3)8:**end while**9:**return **$R^{*},t^{*},\delta d^{*}$

#### 3.2.2. Collision Avoidance

In the iterative surface fitting phase of robot grasp planning, it was observed that while the gripper fingers tend to align toward collision-free grasps, there remains a non-negligible risk of the gripper base colliding with the target object. To mitigate this issue and ensure that the entire gripper body avoids collision, we incorporate a collision avoidance mechanism within the grasp optimization framework.

**Figure 5:**
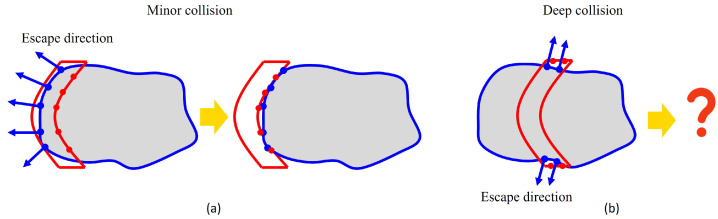
(**a**) In instances of minor collisions, where the interaction between the gripper and the object is relatively superficial, escaping collisions is typically straightforward. (**b**) In situations involving deep collisions, the complexity of avoiding collisions escalates significantly.

An existing method in this domain is the Signed Distance Function (SDF), which computes the distance between each point in a three-dimensional space and the surface of its nearest obstacle. This function offers a differentiable representation of the environment, which proves advantageous for algorithms based on gradient optimization. However, a critical limitation of the SDF approach is its inability to accurately determine the exact penetration vector in scenarios involving deep collisions, as shown in Figure 5. To address collision detection and avoidance, the Gilbert–Johnson–Keerthi (GJK) algorithm coupled with the Expanding Polytope Algorithm (EPA) [33] is commonly employed. The GJK algorithm is utilized to rapidly determine whether a collision occurs through the concept of the Minkowski difference. Subsequent to this, the EPA is tasked with calculating the most efficient vector for collision escape, based on the findings of the GJK algorithm. Despite their utility, these algorithms exhibit limitations, particularly in handling objects with concave geometries. In light of these challenges, particularly prevalent in internal grasp scenarios, we propose a new approach to deep collision avoidance for grasp planning. This approach is designed to effectively navigate the intricacies of deep collision scenarios, thereby enhancing the robustness and reliability of robot grasping.

**Figure 6:**
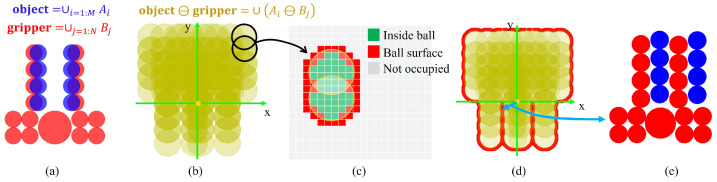
(**a**) Approximate both the object and the gripper into spherical components. (**b**) Calculate the Minkowski difference to formulate a union of spheres. (**c**) Determine the boundary of the union of spheres. (**d**) Find the shortest escape vector. (**e**) The result of the collision-free grasp.

The proposed approach for determining the shortest escape vector in robot grasping scenarios, as depicted in Figure 6, is structured into three steps.

**Figure 7:**
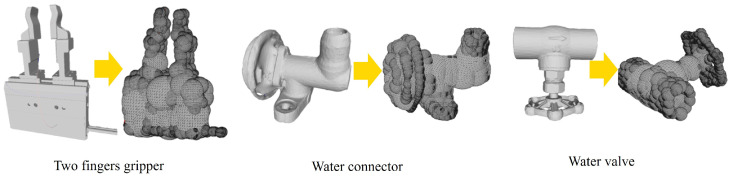
Examples of approximation into spherical components.

The first step involves representing both the object and the gripper as multiple spheres, as shown in Figure 7. This step enables the decomposition of any non-convex object into convex shapes, facilitating the efficient calculation of the Minkowski difference for spherical forms.

Next, the Minkowski difference between each pair of spheres—one from the object and one from the gripper—is computed. This calculation, guided by Equation (10), results in the formation of a union of spheres, collectively representing the overlapping regions between the object and the gripper.

(10) $$P⊖G=\cup \{A_{i}⊖B_{j}|A_{i}\in P,B_{j}\in G\}$$

The object, denoted as *P*, is represented as $P=\cup_{i=1:M}A_{i}$, where each $A_{i}$ signifies an individual sphere on the object. Here, *M* is the total number of spheres that constitute the object. Similarly, the gripper, denoted as *G*, is represented as $G=\cup_{i=1:N}B_{i}$, with each $B_{j}$ representing a sphere on the gripper, and *N* being the total number of spheres that constitute the gripper. The Minkowski difference between two spheres is calculated using Equation (11).

(11) $$A_{i}⊖B_{j}=Sphere\left(c_{A_{i}}-c_{B_{j}},r_{A_{i}}+r_{B_{j}}\right)$$

where $c_{A_{i}}$ represents the center position, and $r_{A_{i}}$ denotes the radius of the sphere $A_{i}$ constituting the object *P*. Similarly, $c_{B_{i}}$ is the center position, and $r_{B_{i}}$ signifies the radius of the sphere $B_{i}$ on the gripper *G*.

The final step involves calculating the boundary of the union of these spheres. The shortest distance from this boundary to the origin is identified as the escape vector. To streamline this process, we introduce a grid-based method to reduce computational complexity. Each grid point is evaluated to determine whether it is inside a sphere, on the sphere’s surface, or unoccupied, according to Equation (12). The shortest escape vector is then determined by identifying the shortest distance between a point on the surface of the combined spherical union and the origin. The vector from this point to the origin represents the shortest possible path to disengage the gripper from the object without collision.

(12) $$\begin{array}{cc} \; & d_{ij}=\left|p_{k}-c_{ij}\right|-r_{ij}\\ \; & \left\{\begin{array}{cc} \; & d_{ij}<0,\;\mathrm{inside}\;\mathrm{ball}\\ \; & d_{ij}=0,\;\mathrm{on}\;\mathrm{ball}\;\mathrm{surface}\\ \; & d_{ij}>0,\;\mathrm{not}\;\mathrm{occupied} \end{array}\right. \end{array}$$

The position of the *k*th grid in the space is denoted by $p_{k}$. $c_{ij}=c_{A_{i}}-c_{B_{j}}$, which represents the center of the union ball $A_{i}⊖B_{j}$, calculated as the difference between the centers of $A_{i}$ and $B_{j}$. $r_{ij}=r_{A_{i}}+r_{B_{j}}$, which is the radius of the union ball $A_{i}⊖B_{j}$, determined by the sum of the radius of $A_{i}$ and $B_{j}$.

## 4. Experiments

In this section, both simulations and real-world experiments are conducted to verify the effectiveness of the proposed LfD-based grasp-planning algorithm. The desktop computer we used was equipped with a 3.7 GHz CPU and 32 GB of memory. An SMC parallel gripper was used in the experiments. The width $d_{1}$ of the gripper finger was 2 cm, whereas the length $d_{2}$ was 10 cm. To incorporate a margin of flexibility in the grasp planning, a scale factor α was introduced, set at 1.5. Applying this scale factor, the parameters of the gripper were adjusted to $d_{1}=\alpha \cdot 2=3$ cm and $d_{2}=\alpha \cdot 10=15$ cm.

### 4.1. Experiments through Simulations

**Figure 8:**
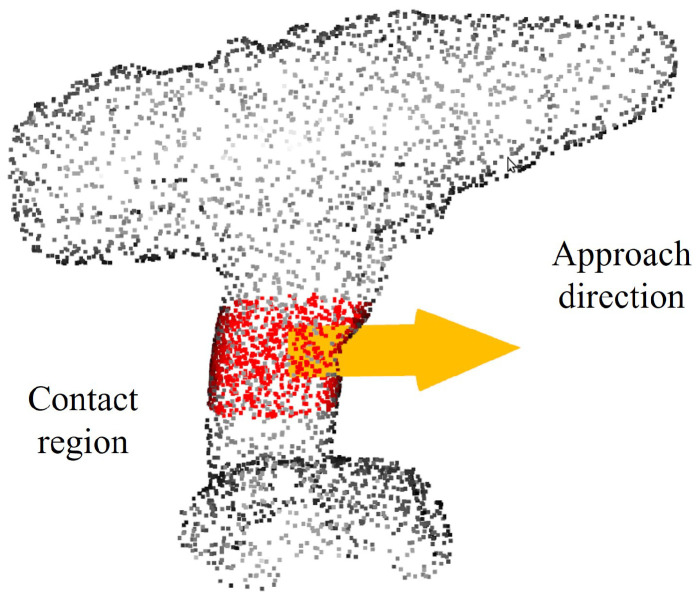
The red area is the contact region (red) and the yellow arrow is the approach direction.

**Figure 9:**
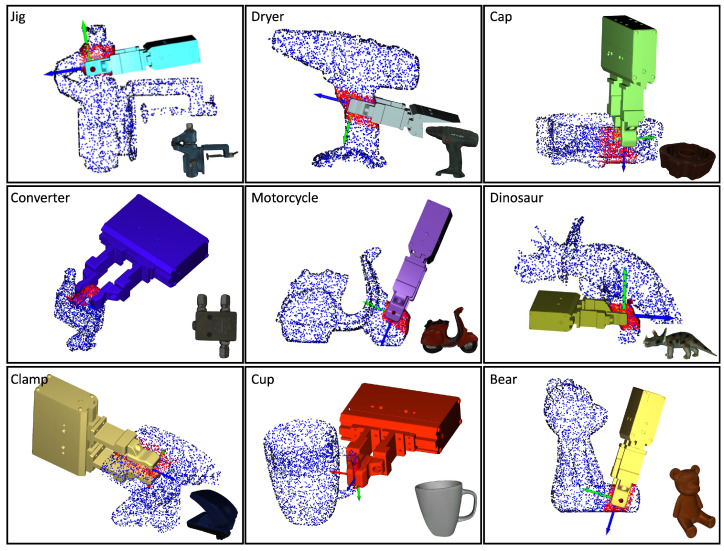
The simulation results on different objects. The nine objects were selected from the HomebrewedDB dataset.

**Figure 10:**
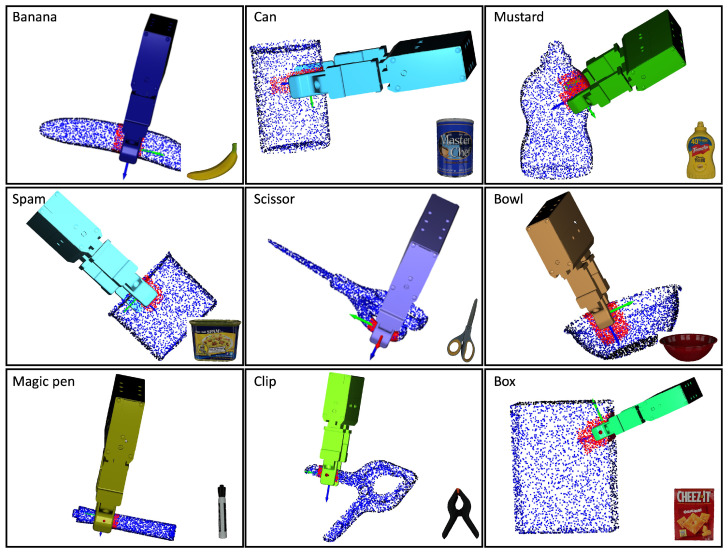
The simulation results on different objects. The nine objects were selected from the YCB-Video dataset.

**Figure 11:**
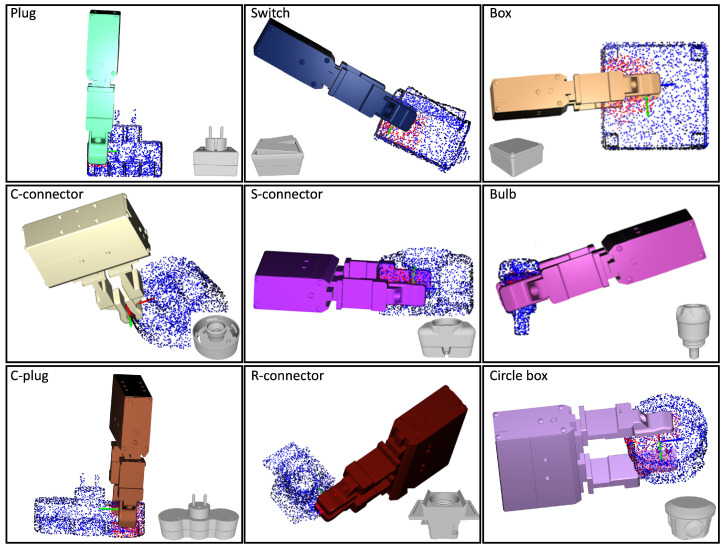
The simulation results on different objects. The nine objects were selected from the T-less dataset.

In the simulations, the contact regions and the approach direction were specified by the human operator through the use of a mouse, as shown in Figure 8. The method employed for evaluation incorporated three datasets: HomebrewedDB [34], YCB-Video [35], and T-less [36]. To ensure a comprehensive and varied analysis, nine objects were picked from each dataset, resulting in a diverse range of 27 objects. To evaluate the proposed method, the original iterative surface fitting (ISF) method was employed as a benchmark. The evaluation metric was the surface fitting error $E_{total}=E_{p}+\lambda E_{n}$, as defined by Equation (3). This metric quantifies the average distance, in millimeters, between the corresponding points on the surfaces of the gripper tips and the object, thus providing a measure of the accuracy of the fit. In addition, the computation time for grasp planning, measured in seconds, was recorded for each method. The simulated results for the various objects are presented in Figure 9, Figure 10 and Figure 11. Furthermore, as detailed in Table 1, the simulation results revealed certain limitations of the original ISF method, particularly its tendency to converge to local optima, requiring the resampling of grasp poses. This resampling process subsequently resulted in increased computation times. In contrast, the proposed method, incorporating contact regions and approach directions specified by human operators, demonstrated improved performance. Specifically, it achieved reduced fitting errors and shorter computation times for determining the optimal grasp poses owing to the employment of closed-form solutions for grasp planning. The experimental results underscore the enhanced efficiency and accuracy of the proposed method in grasp-planning applications.

**Figure 12:**
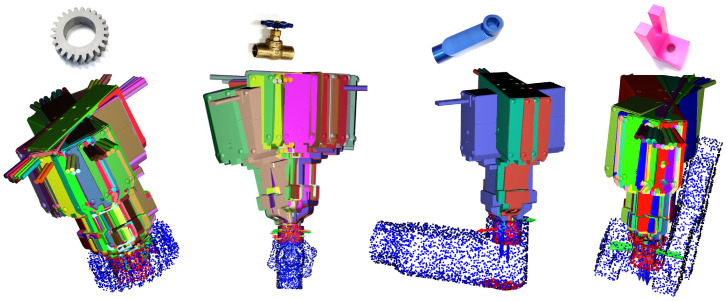
The simulation results of the internal grasping of different objects.

We further evaluated the effectiveness of the proposed collision avoidance method in scenarios involving the internal grasping of four distinct objects. The results are visualized in Figure 12. The process involved randomly sampling an initial grasp pose, followed by the generation of collision-free grasp poses using the surface fitting method. The evaluation metric was the success rate of collision-free optimal grasps based on a total of 20 samples, comparing scenarios with and without the implementation of collision avoidance. The comparison results are detailed in Table 2. It is noteworthy that internal grasping presented a significant challenge due to the necessity of locating a feasible grasp within a narrow space, a task that is markedly more complex in the absence of a collision avoidance mechanism. Our findings demonstrate that integrating the collision avoidance function significantly enhanced success rates across all four objects.

### 4.2. Experiments with a Real Robot

The proposed approach was further validated on a FANUC LR-Mate 200iD (FANUC America Corporation, Rochester Hills, MI, USA), a 6-degree-of-freedom industrial robot, tested with various objects. For detecting human hand movements, an Intel RealSense D435 camera (Intel Corporation, Santa Clara, CA, USA) was utilized in these experiments. Compared with other sensors, such as data gloves or thermal cameras, the advantage of using a 3D camera is that it allows for the same sensor to be used for human hand tracking in human demonstration and object detection in robot execution since the demonstrator and the robot share the same workspace. The well-known Iterative Closest Points (ICP) method was employed for object localization. After detecting the positions of both the human hand pose and the target object, the contact region and approach direction were calculated within the camera coordinate system. Once the optimal grasp was determined in the camera coordinates, the result was subsequently transformed into the robot coordinate system for execution by the robot.

**Figure 13:**
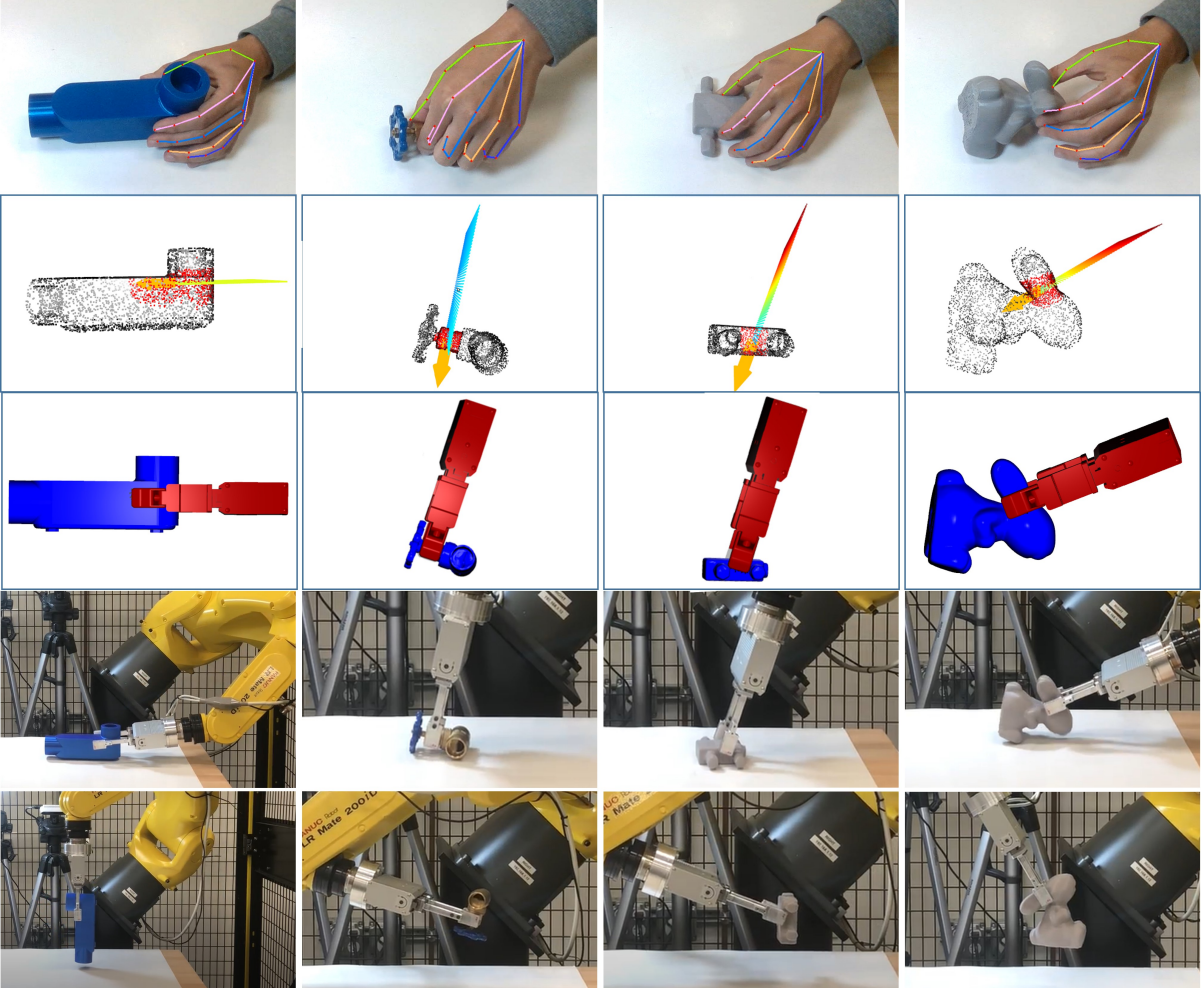
The results using a real robot. After lifting the objects, the robot rotates the objects 90 degrees to simulate a post-grasp motion.

The proposed LfD-based grasp planning was demonstrated on four different objects: a water pipe, water valve, converter, and toy rabbit. The results are shown in Figure 13. The top row of the figure illustrates the human demonstration. The second row shows the hand plane; the identified contact region, marked in red; and the approach direction, marked in yellow. The third row shows the generated optimal grasp, whereas the fourth row shows the robot successfully executing the grasp. Finally, the bottom row illustrates the ability of the proposed method to rotate the object by 90 degrees relative to the ground, simulating a pose-grasp motion. The experimental videos are available in Supplementary Materials.

**Figure 14:**
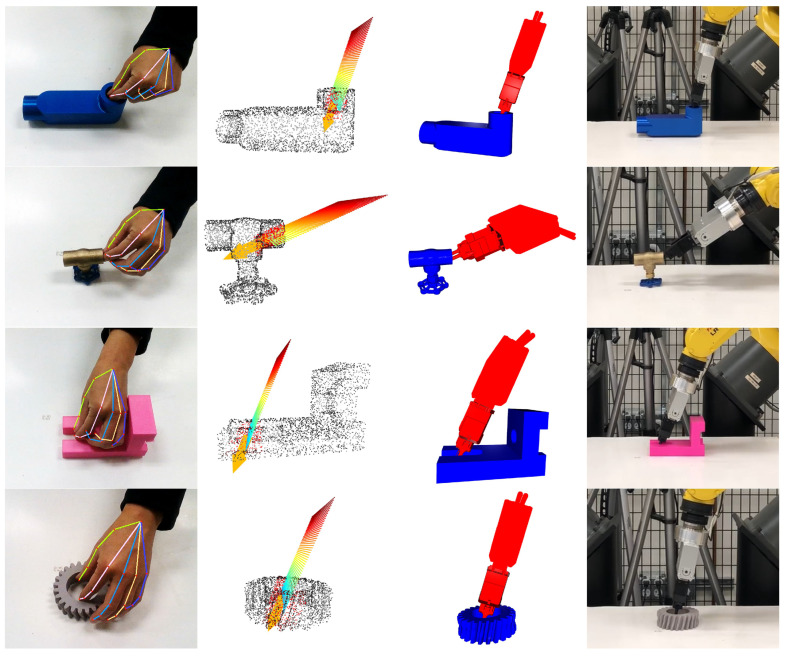
The experimental results of the internal grasp case using a real robot.

We also evaluated the proposed method to encompass scenarios involving the internal grasping of four distinct objects. The results, as illustrated in Figure 14, affirm that our method is capable of identifying feasible grasping poses, even within restricted spatial confines. This is primarily attributed to the integrated collision avoidance function, which effectively identifies an escape vector to facilitate the generation of a collision-free grasp.

To quantitatively evaluate the proposed method, we conducted a series of experimental comparisons: Method 1 employed only the contact region; Method 2 employed only the approach direction; and Method 3 utilized both the contact region and approach direction. The evaluation metric was the success rate, indicating the robot’s ability to reach the grasp pose generated from human demonstrations without colliding with the surrounding environment. The results of these experiments are comprehensively detailed in Table 3. Note that only Method 1 and Method 3 were subjected to comparative analysis and testing in the case of internal grasp since it was necessary to specify the contact region for generating an internal grasp. The analysis revealed that Method 1 exhibited the lowest success rates. This outcome can be attributed to the absence of approach direction information, leading to frequent collisions with the ground during grasp attempts. This indicates the critical importance of approach direction in facilitating a functional grasp. Method 2 demonstrated better success rates compared to Method 1. Notably, the integration of the approach direction reduced the frequency of collisions with the surrounding environment. However, the lack of contact area information often resulted in grasping poses tending toward an unstable position, hindering the successful lifting of the object. In contrast, Method 3 exhibited the highest success rates, suggesting that the inclusion of both the contact region and approach direction was effective. It was observed that the incorporation of a good initial contact region and approach direction derived from human demonstrations significantly expedited the convergence of the optimization algorithm to a feasible solution.

These experimental results not only validate the proposed method but also highlight its practical applicability in real-world grasp-planning scenarios. Despite the diverse shapes of the target objects, the proposed LfD-based approach demonstrated its efficiency in synthesizing human-like grasps. A key strength of this approach lies in its ability to adeptly avoid collisions, a critical aspect in robotic grasping scenarios. Furthermore, the method ensures a firm grasp of the object, which is essential for successfully executing post-grasp motions. This adaptability to various object geometries while maintaining a reliable and collision-free grasp underscores the robustness of the proposed method. The effectiveness of replicating human-like grasping strategies enhances the practical applicability of robotic systems in diverse environments.

## 5. Conclusions

This work introduces a learning from demonstration-based grasp-planning approach that effectively generates stable grasps by integrating grasp skills derived from human demonstrations. We demonstrate that the proposed method can intuitively acquire critical grasp skills, namely the contact region and approach direction, from a single human demonstration using a standard RGB-D camera. This approach contrasts with and offers advantages over more specialized equipment such as data gloves or thermal cameras. In addition, the optimal grasp is solved using a closed-form solution, which incorporates the nuances of human grasp skills. To validate the effectiveness of this approach, a series of experiments is conducted using a FANUC industrial robot. The results from these experiments demonstrate that the proposed method not only achieves stable grasping but also effectively aligns with human intentions for post-grasp motions.

In the future, we plan to extend the algorithm to accommodate multi-finger grippers while considering the kinematic constraints of such grippers. In addition, there is an interest in exploring how general grasp skills can be extracted from human demonstrations across a variety of object shapes. This exploration is expected to leverage deep learning techniques, potentially leading to more versatile and adaptable robot grasping capabilities.

## Figures and Tables

**Table 1:** Surface fitting errors and computation times on different objects.

Dataset	Object Name	ISF		LfD-Based ISF	
		$E_{\mathrm{total}}$ (mm)	$t_{\mathrm{total}}$ (s)	$E_{\mathrm{total}}$ (mm)	$t_{\mathrm{total}}$ (s)
HomeDB	Jig	13.005	1.191	5.326	0.798
	Dryer	14.600	1.071	6.974	0.747
	Cap	9.609	1.990	5.459	0.975
	Converter	11.387	2.561	6.404	0.519
	Motocycle	12.032	2.439	7.285	0.661
	Dinosaur	17.219	1.744	9.427	0.895
	Clamp	9.439	1.240	7.395	0.614
	Cup	10.871	1.867	8.068	0.771
	Bear	12.758	1.043	7.215	0.558
	Average	12.324	1.683	7.061	0.726
YCB-V	Box	3.457	0.404	3.560	0.369
	Can	4.193	0.420	2.551	0.406
	Mustard	8.720	0.832	6.174	0.559
	Spam	8.740	0.532	4.283	0.370
	Banana	3.145	0.434	3.031	0.287
	Bowl	8.924	1.238	4.778	0.862
	Scissor	5.808	0.679	3.662	0.277
	Magic pen	4.806	0.523	4.092	0.317
	Clip	6.836	0.379	4.681	0.347
	Average	6.070	0.604	4.090	0.422
T-less	Bulb	5.198	0.506	2.600	0.327
	S-connector	8.772	0.872	3.588	0.334
	C-plug	6.968	1.004	6.525	0.532
	R-connector	11.648	1.216	7.858	0.524
	C-connector	10.593	1.562	6.623	0.639
	Switch	5.159	0.915	3.409	0.359
	Box	2.405	0.439	2.536	0.412
	Plug	13.058	1.282	5.171	0.865
	Circle box	7.784	0.526	5.417	0.329
	Average	7.954	0.925	4.859	0.480
Total average		8.783	1.071	5.337	0.543

**Table 2:** Success rates (collision-free grasps/total samples), with and without the implementation of collision avoidance.

Object Name	Gear	Water Valve	Water Pipe	Jig
Without collision avoidance	11/20	13/20	10/20	7/20
With collision avoidance	20/20	18/20	19/20	20/20

**Table 3:** Success rates (collision-free grasps/total samples).

	External Grasp				Internal Grasp			
Objects	Water Pipe	Water Valve	Converter	Toy Rabbit	Gear	Water Valve	Water Pipe	Jig
Method 1	11/20	6/20	2/20	13/20	11/20	7/20	17/20	12/20
Method 2	15/20	10/20	13/20	13/20	-	-	-	-
Method 3	18/20	20/20	20/20	19/20	20/20	20/20	20/20	20/20

## Data Availability

Data are contained within the article.

## Supplementary Materials

The following supporting information can be downloaded at: https://www.mdpi.com/article/10.3390/s24020618/s1, Video S1: Experimental Videos for Learning from Demonstration-Based Grasp Planning.
